# Green Analytical Method Using Single-Drop Microextraction Followed by Gas Chromatography for Nitro Compound Detection in Environmental Water and Forensic Rinse Water

**DOI:** 10.3390/molecules30091894

**Published:** 2025-04-24

**Authors:** Tamara Pócsová, Senad Okanovič, Svetlana Hrouzková

**Affiliations:** Faculty of Chemical and Food Technology, Institute of Analytical Chemistry, Slovak University of Technology in Bratislava, Radlinského 9, 812 37 Bratislava, Slovakia; tamara.pocsova@stuba.sk (T.P.);

**Keywords:** nitro compounds, forensic samples, environmental water, single-drop microextraction, gas chromatography

## Abstract

The extensive use of nitro compounds in agriculture, industry, armaments, and pharmaceuticals, along with their toxic effects on living organisms, necessitates efficient and environmentally sustainable analytical methods. Traditional extraction techniques often involve practices that are not eco-friendly, such as the use of large volumes of solvents, toxic chemicals, and the generation of significant waste; therefore, the single-drop microextraction technique was involved in overcoming these limitations. This study shows an environmentally friendly method for nitro compound analysis focusing on NB (Nitrobenzene), 2-NT (2-Nitrotoluene), 3-NT (3-Nitrotoluene), 4-NT (4-Nitrotoluene), 1,3-DNB (1,3-Dinitrobenzene), 1,2-DNB (1,2-Dinitrobenzene), 2,4-DNT (2,4-Dinitrotoluene), and TNT (Trinitrotoluene). To separate and to detect selected nitro compounds, gas chromatography with an electron capture detector was utilized, which is highly selective for analytes containing nitro groups. To determine optimal experimental conditions, extraction parameters were studied, including the impact of salt addition, temperature, and pH on extraction efficiency. Key performance parameters, such as limit of detection (LOD), limit of quantification (LOQ), repeatability, extraction recoveries, calibration range, and matrix effects, were assessed. The LOD values ranged from 0.01 to 0.09 μg/L in deionized water, 0.01 to 0.06 μg/L in tap water, 0.01 to 0.03 μg/L in seawater, and 0.03 to 0.11 μg/L in model forensic rinse water. The optimized method was successfully applied to the determination of nitro compounds in real environmental water samples and forensic rinse water samples. The environmental sustainability and greenness of the proposed method was evaluated with the AGREE, AGREEprep, and AESA techniques.

## 1. Introduction

Nitroaromatic compounds are among the largest and most important groups of industrial chemicals currently in use. These compounds are organic molecules that consist of at least one nitro group (-NO_2_) attached to an aromatic ring. The vast majority of these are synthetic, although a few biologically produced nitroaromatic compounds have been identified. Nitroaromatic compounds can occur naturally in both atmospheric and aqueous environments. In the urban environment, hydrocarbons released from natural combustion processes and the incomplete combustion of fossil fuels serve as substrates for nitration with atmospheric nitrogen dioxide [1]. In the aquatic environment, solar radiation catalyzes the nitration and halogenation reactions of naturally occurring or anthropogenic compounds in a similar manner [2]. Although the vast majority of nitroaromatic compounds are synthesized chemicals, they have also been discovered as natural products from various bacteria, fungi, and plants [3]. Although nitroaromatic compounds that naturally occur in the environment are relatively rare, they have mainly entered the environment through human activity. This important class of industrial chemicals is used in large volumes in the synthesis of a variety of products including explosives, dyes, polymers, and pesticides. Unfortunately, their widespread use has led to the contamination of soil and groundwater in the environment. The nitro group, which provides the chemical and functional diversity of these molecules, also contributes to the resistance of these compounds to biodegradation. Nitroaromatic compounds are dangerous to human health due to their toxicity, mutagenicity, and easy reduction to carcinogenic aromatic amines [1]. Although some nitroaromatic compounds are intentionally applied to the environment (pesticides), improper handling and/or storage practices by manufacturers and users have led to their accidental release into the environment in countries around the world. The annual tonnage of released chemicals reflects the enormous scale of this problem [4]. Industrial accidents have also led to the contamination of the environment with nitroaromatic compounds. The most significant contamination in recent history occurred on 4 August 2020 in Beirut, Lebanon, where an ammonium nitrate warehouse exploded [5]. Another major environmental contamination occurred at a nitration unit during a chemical production plant explosion in Jilin, China, and about 100 tons of benzene and nitrobenzene leaked into the Songhua River [6]. Explosives are reactive substances that, upon initiation, undergo a rapid chemical transformation which produces gas, heat, and pressure, leading to an explosion. Explosive detection methods have been designed for various applications, including humanitarian demining, addressing environmental concerns like groundwater and soil remediation (as explosive residues pose a risk to human health), security screening, intelligence operations, criminal forensics, and rapid sample analysis, or the quantification of nitroaromatic compounds [7]. Another source of pollution is cars and exhaust fumes. Unfortunately, their widespread use has led to the contamination of soil and groundwater in the environment.

Chromatographic methods are often used to determine and separate nitro compounds in environmental matrices (most often water and soil samples). Mainly gas chromatography methods coupled with mass spectrometry (GC-MS) or an electron capture detector (GC-ECD) are suitable for the analysis of non-polar and volatile nitro compounds. Due to the incompatibility of most gas chromatography columns with water samples and due to the need to concentrate the analytes, it is necessary to choose a suitable extraction technique for extracting the analytes from the matrix.

To treat the water sample from environmental sources, conventional techniques such as Solid Phase Extraction (SPE) [8,9], Dispersive Solid Phase Extraction (dSPE) [10], Solid Phase Microextraction (SPME) [11,12,13,14], Switchable Hydrophilicity Solvent-Based Microextraction (SHS-ME) [15], Microextraction by Packed Sorbent (MEPS) [16,17], Stir Bar Sorptive Extraction (SBSE) [18], Dispersive Liquid–Liquid Microextraction (DLLME) [19,20,21], Single-drop Microextraction (SDME) [22,23,24], and Hollow Fiber Solid–Liquid Phase Microextraction (HF-SLPME) [12,24,25] were used. SDME is an attractive extraction technique mainly because of its low cost and ease of use. Finally, it follows the principles of green analytical chemistry, as only a few microliters of the extraction solvent are consumed for one extraction. The most commonly used SDME techniques include Headspace Single-drop Microextraction (HS-SDME), in which the extraction solvent does not come into direct contact with the sample but is suspended on the tip of a microsyringe in the “head-space” phase above the liquid sample surface [26,27,28,29], and Direct Immersion Single-drop Microextraction (DI-SDME), where the microdrop of the extraction solvent is directly immersed in the liquid sample [20,21,22,23,24,25,26,27,28,29,30,31,32]. As an extraction solvent, toluene [23,31], chlorobenzene [30], butyl acetate [26], n-octanol [32], N,N-dimethylformamide [28], or their combination, specifically toluene and butyl acetate [33] and benzyl alcohol and ethyl acetate [24] were already reported. A modern trend in SDME techniques is to replace traditional organic solvents and chlorinated solvents, such as chlorobenzene or chloroform, with greener alternatives with less impact on the environment [22,27,29]. Ecological solvents for microextraction techniques are a relatively new topic. New and innovative applications of deep eutectic solvents (DESs), switchable hydrophilicity solvents (SHSs), and supramolecular solvents (SUPRASs) in sample preparation are gaining increasing attention [34].

The main aim of the presented study is to develop a green analytical method based on SDME connected with GC and ECD detection for the simultaneous determination of nitro compounds from various types of environmental water and forensic rinse water samples. In this paper, the authors aim to present the optimized extraction method for different types of water samples and to optimize conditions for the variability of matrices. To our knowledge, detailed optimizing microextraction conditions and study of matrix effects for the analysis of nitro compounds for forensic rinse water samples have not been published so far.

## 2. Results and Discussion

For liquid phase samples, such as various types of environmental water and forensic rinse water, direct liquid extraction of nitro compounds is a promising sample pretreatment technique. Nowadays, there is a great emphasis on the greenness of the methods used; therefore, the minimization of extraction reagent leading to liquid microextraction techniques is a suitable option for testing the possibilities of analysis performed in a greener manner. The samples potentially contaminated with eight nitro compounds were analyzed, and the DI-SDME was used for extraction. Using the SDME technique, several extraction conditions have an important impact on the analytical method and the validation parameters, such as the choice of solvent, its volume, extraction time, the choice of mixing conditions, sample pH, and ionic strength.

### 2.1. Selection of Extraction Conditions

#### 2.1.1. Selection of the Extraction Solvent and Its Volume

Working with aqueous solutions as samples, it is essential that the extractive solvent is immiscible with water so that it can form a microdrop in water. Several solvents were tested. Hexane as the extraction solvent is immiscible with water, but it was not able to form a stable microdrop in water. Of the potentially green solvents, n-butyl acetate was tested, as it is immiscible with water. This solvent created a stable microdroplet on the tip of the microsyringe. Unfortunately, during the extraction process, the volume of the microdroplet decreased, and after the end of the extraction, only a small fraction of the original volume of the droplet could be obtained as the extract. A volume of 0.2 µL was obtained from the original 1 µL n-butyl acetate drop; therefore, the solvent was not further applied. The next solvent tested was toluene. Toluene is immiscible with water and was able to form a stable microdroplet that maintained its original volume throughout the extraction time. The use of binary extraction solvents, which are mixtures of two extraction solvents, affects the polarity, density, and solubility of the final extraction product and thus affects the efficiency of the entire extraction procedure. In the next phase of testing, binary solvents were tested. Hexane and n-butyl acetate, respectively, were mixed with toluene in different proportions to eliminate the shortcomings of the listed solvents used individually. In the case of mixing hexane and toluene, two liquid immiscible phases that were not useful for the extraction were obtained. Combining n-butyl acetate with toluene produced a single liquid phase; unfortunately, the binary solvent droplet composition showed significant droplet volume loss during the extraction. A binary microdrop composed of toluene and n-butyl acetate in a ratio of 1:1 with a volume of 3 µL lost approximately one-third of its original volume during the extraction process. Therefore, only toluene was used as the extraction solvent in further experiments.

**Figure 1:**
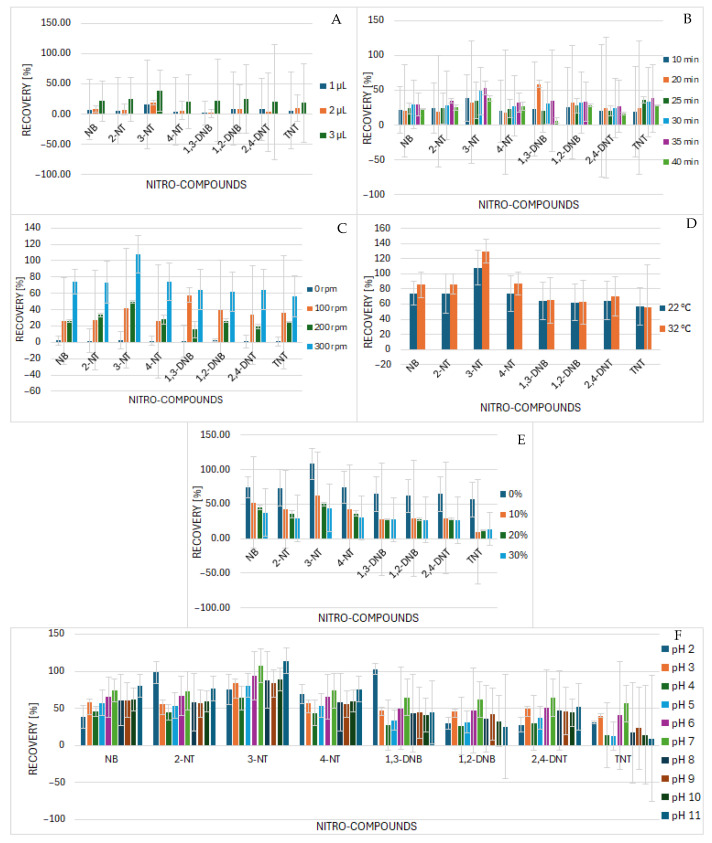
Dependence of the recovery of individual nitro compounds on the (**A**) volume of the microdrop, (**B**) the time of the extraction, (**C**) the stirring intensity of the extraction process, (**D**) the extraction temperature, (**E**) the addition of NaCl, and (**F**) the change in pH.

The droplet surface, through which the transfer of analytes from the sample to the extraction solvent occurs, is directly influenced by the quality of the solvent. The highest surface of the droplet will be increasing with the increase in the volume of the solvent. The calculated values of the vapor volume of the extraction solvent and the ability of the droplet to stay on the tip of the microsyringe throughout the extraction time were studied. For a 3 µL microdrop of toluene, under the selected pressure and temperature conditions of the subsequent GC analysis, the vapor volume was 442 µL for the given split/splitless inlet arrangement with the single-tapered liner type. The volume of the single tapered liner is 900 µL, so filling it with the injected extract to about half is suitable for injection to the chromatographic system. A drop with a volume greater than 3 µL did not remain on the microsyringe during the entire extraction time and would not be suitable for injection at the given inlet setting and temperature program; therefore, extractions were tested with drops at volumes of 1, 2, and 3 µL, while the extraction time remained constant at 10 min. Figure 1A shows the dependence of analyte recoveries on the droplet volume at the fixed extraction time of 10 min. For all analytes, by increasing the droplet volume, higher recovery values were obtained. Figure 1 also reveals the fundamental problem of SDME under the given conditions, which is repeatability. It was necessary to search conditions through optimization so that the system will be repeatable and robust.

#### 2.1.2. Selection of the Time of the Extraction

The extraction time is the time during which the analytes are extracted into the extraction solvent. Too short an extraction time can lead to an incomplete transfer of analytes and thus significantly reduce the efficiency of the method. Conversely, too long an extraction time can lead to the deformation of the drop or its partial dissolution in the water sample. Therefore, it was necessary to choose an appropriate extraction time between these two extreme situations. The optimal time of the extraction was tested between 10 and 40 min. The highest peak areas of tested analytes were obtained using the extraction time of 35 min. Therefore, it was selected for further studies. The lower recoveries (also peak areas of analytes) in the 40 min extraction were caused by the accumulation of air bubbles in the droplet, which caused a part of it to break off and did not allow it to recover its entire volume. The intensity of mixing and the time the drop spent in the water could have contributed to the formation of tiny bubbles. All analytes showed a trend of increasing recoveries with increasing extraction time up to 35 min. Isolated drops could be caused by the break-off of a small volume of the drop or the formation of bubbles. Figure 1B shows the dependence of the recovery of tested analytes on the time of the extraction.

#### 2.1.3. Selection of Stirring Intensity

The solution must be stirred throughout the extraction process to achieve a better transfer of analytes into the extraction microdrop. The optimization of this step is extremely important, because it is a micro-scale extraction, and the volume of the solvent is small, and it is placed in the fixed position in the sample throughout the extraction. It was expected that with an increase in the intensity of stirring, the peak areas of the analytes would also increase. The intensity of stirring was tested at the stirring rates between 0 and 400 rpm. At no stirring conditions (0 rpm), minimal recoveries (as well as peak areas of the analytes) were recorded, and at stirring intensity above 400 rpm, the droplet did not stay on the microsyringe tip for the entire extraction time and fell from the microsyringe tip. The highest recoveries (peak areas) were achieved at 300 rpm; the drop was stable during the entire extraction, and it was possible to load back its entire volume. By increasing the stirring intensity, a better mixing of the solution was achieved, but it led to the accelerated formation of bubbles in the drop, and thus, it was not always possible to load the entire volume of the drop back into the microsyringe. The repeatability of the experiments at the stirring intensity of 100 rpm was not acceptable due to high RSDs, which is mainly due to the instability of the drop at the needle tip. Figure 1C shows the dependence of the recovery of tested analytes on the stirring intensity. Further experiments were performed at 300 rpm.

#### 2.1.4. Selection of the Extraction Temperature

Increasing the temperature leads to greater solubility of the analytes in the sample but also to a decrease in the viscosity of the extraction solvent. In DI-SDME, this contributes to a higher instability of the drop, which causes a partial loss of volume due to its mixing with the aqueous phase or its complete detachment. The extraction temperature was examined at 22 °C, 32 °C, and 42 °C ± 1 °C. It was assumed that the transfer of analytes into the extraction solvent would improve with increasing temperature, which was confirmed through experiments. At a temperature of 32 °C, slightly higher analyte recoveries were achieved in comparison to experiments at the laboratory temperature. However, at an elevated temperature of 32 °C, it was not possible to regularly and reliably load back the entire volume of the drop into the microsyringe, and there was also an increased formation of tiny bubbles in the drop, which led to a worsening of the repeatability as well as success of the extraction. On average, only one out of four extractions were successful. In all other cases, the microdrop fell down from the microsyringe needle tip. At a temperature of 42 °C, a microdrop of the extraction solvent was not able to stay on the tip of the microsyringe during the entire extraction time. This phenomenon can be attributed to the fact that with increasing temperature, the viscosity of toluene decreases, and its mixing with the aqueous phase occurs, as well as the increased formation of bubbles in the drop. Satisfactory RSD values (15–26%) were achieved at 22 °C, which we attribute to less bubble formation in the drop and in the water sample, as well as a higher viscosity of the extraction solvent, which led to an increase in its stability. The highest extraction recoveries (peak areas of studied analytes) were achieved at 32 °C. The extraction temperature we continued to work with was 22 °C, although with lower analyte peak areas of the analytes, which resulted in a much higher extraction success rate of performing the extraction without a drop falling off the microsyringe needle tip; thus, the repeatability and robustness of the method was the highest (Figure 1D).

#### 2.1.5. Addition of Salt

The addition of salt to the sample increases the ionic strength, which affects the solubility of organic analytes. The salt effect occurs, causing water molecules to interact with salt ions. Theoretically, this interaction disrupts the hydrogen bridges formed by the analytes, thereby increasing the transition of the analytes from the aqueous phase to the organic phase. The extractions were performed without the addition of salt and then with the addition of NaCl at concentrations of 10%, 20%, and 30% (*w*/*v*). The highest extraction recoveries were achieved without the addition of salt. Figure 1E shows the effect of NaCl addition on the recovery of individual nitro compounds. The graph shows that the highest recoveries were achieved in extractions without the addition of salt, while the lowest recoveries were achieved with the increase in the addition of salt. Also, less droplet stability after salt additions was observed, leading to reduced extraction success. No addition of NaCl was selected for the DI-SDME in the next studies.

#### 2.1.6. Selection of the Working pH

Waters of different origins have different pH values. The deionized water that was used during the extraction had a pH value of 6. In contrast, sea water had a pH value of approximately 8, while mineral water and bottled water intended for consumption have pH values in the range of 5 to 10, depending on the manufacturer and the place of origin of the water. When determining the pH range in which the extractions will be performed, it was assumed that when analyzing real post-explosion products, the pH of the sample may be more acidic or basic than that of environmental water. Therefore, the study with water samples within the values of pH from 3 to 11 was performed. Aqueous solutions of citric acid and KOH were used to adjust the pH. The highest recoveries of analytes were achieved in a water sample with a pH value of 6, i.e., in deionized water without pH adjustment. In principle, significant differences between the extractions performed either in an acidic or alkaline environment were not noticed. The lowest RSD values were recorded for deionized water; however, in an alkaline environment of pH 8 to 11, an increase in the RSD values of extractions compared to the RSD values of extractions in an acidic environment was observed. In conclusion, the precision of the extraction is better in neutral and acidic environments. Therefore, it is necessary to evaluate the results of quantitative analysis for samples outside the optimal pH range by performing method validation for other pHs by using matrix-matched standards (Figure 1F).

### 2.2. Validation Process

The validation of the DI-SDME GC-ECD method was established in terms of linearity, the dynamical liner range, precision, accuracy, the limit of detection (LOD), and the limit of quantification (LOQ). To calculate the validation parameters, three calibration curves in matrices of deionized water, tap water, and seawater were performed. The capacity of the detector cell was no longer sufficient for higher concentrations. For analytes in deionized water, LOD values dependent on the individual nitro compounds ranged from 0.01 to 0.09 μg/L and LOQ values from 0.03 to 0.31 μg/L. The coefficients of determination were in the range of 0.9961 to 0.9994 for all analytes. Analytes in the tap water matrix had LOD values from 0.01 to 0.06 μg/L and LOQ values from 0.03 to 0.19 μg/L. The coefficients of determination were in the range of 0.9977 to 0.9993. For analytes in the seawater matrix, LOD values ranged from 0.01 to 0.03 μg/L and LOQ values from 0.03 to 0.11 μg/L. The coefficients of determination had a value of 0.9974 to 0.9992. For analytes in the model forensic rinse water matrix, the LOD values range from 0.03 to 0.11 μg/L and LOQ values from 0.11 to 0.38 μg/L. The coefficients of determination had a value of 0.9611 to 0.9965. Table 1 and Table 2 shows the validation parameters for different types of water samples.

### 2.3. Study of the Matrix Effect

**Figure 2:**
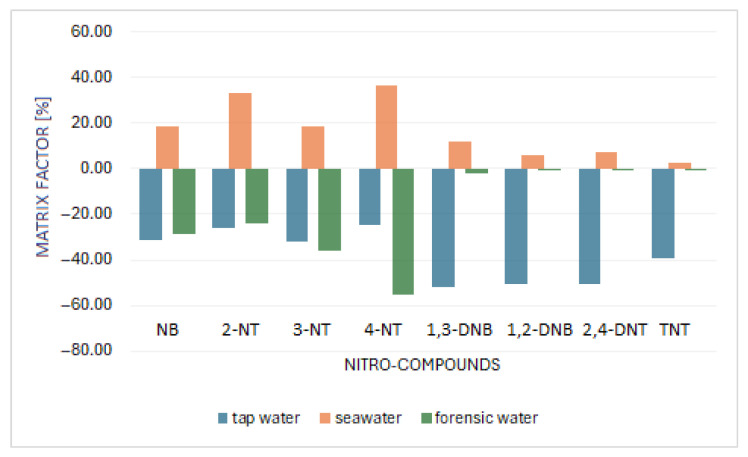
Matrix factors of individual analytes in different types of water samples.

The matrix effect may manifest either an increase or decrease in the detector signal compared to the response from solvent solutions of the analytes. The matrix factor (MF) was calculated for each studied nitro compound by comparing the peak area of the analyte in the matrix-matched solution vs. the peak area of the nitro compound in distilled water. The MF was investigated in tap water, in seawater, and in model forensic rinse water. A positive value of the matrix effect corresponds to an amplification of the matrix-induced signal, while a negative value corresponds to a signal attenuation effect. In general, matrix effects can be classified as weak (0–20%), moderate (20–50%), and strong (>50%). The matrix factors are summarized in Table 3 and Figure 2.

As shown in Figure 2, moderate and high matrix effects were detected for analytes in tap water. Weak matrix effects were detected for most of the analytes in the seawater matrix, with the exception of 2-NT and 4-NT, where the matrix effect was moderate. Moderate matrix effects were found for 3-NT and 4-NT in the model forensic rinse water matrix, and weak matrix effects were found for the other analytes. We can evaluate that for nitrotoluene isomers in all matrices, the matrix effects were significantly lower than those for the other analytes.

### 2.4. Real Sample Analysis

**Figure 3:**
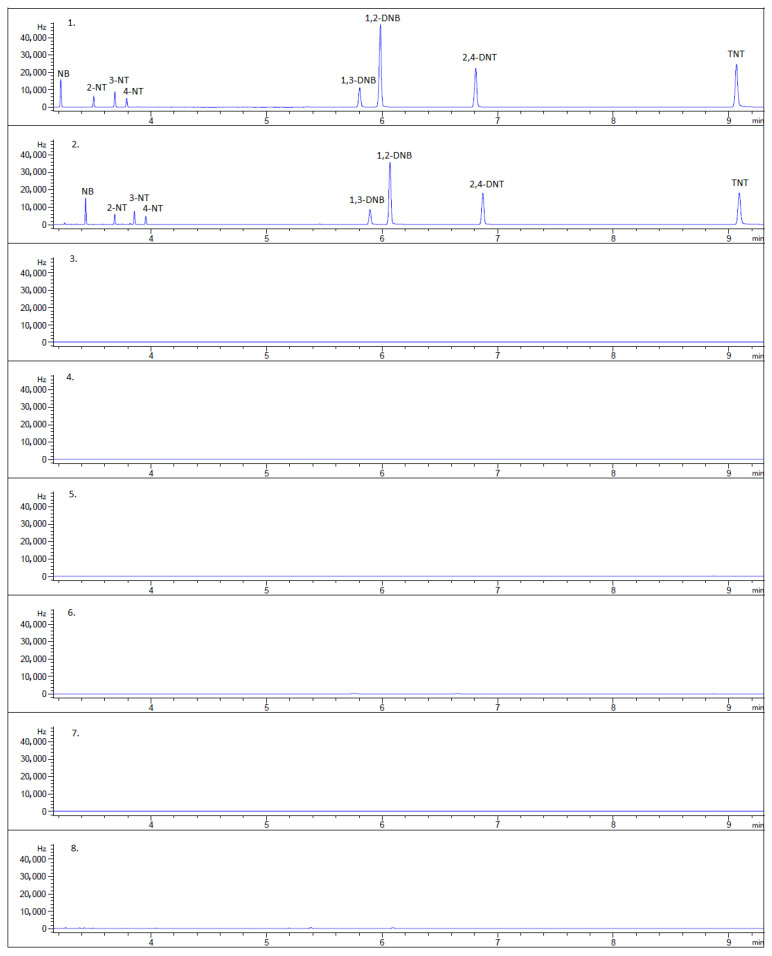
Chromatograms of real samples: (**1**) nitro compounds in the solvent (toluene), (**2**) the spiked water sample with nitro compounds, (**3**) the forensic rinse water sample, (**4**) the seawater sample, (**5**) the drinking water sample, (**6**) the drinking water sample, (**7**) the drinking water sample, (**8**) the drinking water sample.

The applicability of the method was tested via real sample analyses under optimized conditions in various types of water samples: forensic, seawater, and tap water. Four samples of tap water were collected from different points of Slovakia, and one seawater sample and one forensic rinse water sample were analyzed. Figure 3 shows the result of the chromatographic analyses. No levels above the limit of detection were found.

### 2.5. Greenness of the DI-SDME Method

**Figure 4:**
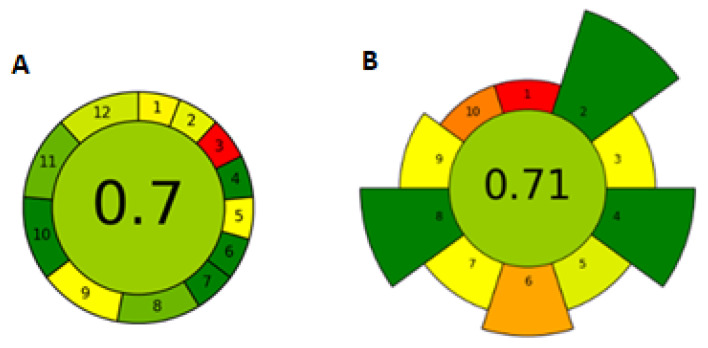
Evaluation of the greenness of the method according to (**A**) AGREE and (**B**) AGREEprep.

The evaluation of the greenness of the method was conducted according to AGREE, an analytical greenness calculator software (https://agree-index.anvil.app/ accessed on 26 March 2025); AGREEprep, an analytical greenness metric for sample preparation (https://agreeprep.anvil.app/ accessed on 26 March 2025); and the analytical eco-scale assessment (AESA) method [35]. The AGREE software consists of twelve questions; AGREEprep is based on 10 categories of green analytical chemistry, and according to the answers, it rates the greenness of the method from 0 to 100% [36,37]. The AESA method deducts points for the use of toxic chemicals, energy consumption, and the amount of waste produced, among others [38]. Figure 4A shows the results of the method evaluation with AGREE.

The resulting evaluation of the greenness of the method according to AGREE was 70%. The main shortcomings identified were off-line sampling and analysis (1, 3), manual injection (5), and GC-ECD as a separation technique. The sample volume was suboptimal for greenness and noted that the method lacked automation. If we consider the evaluation of the parameters related exclusively to the extraction method, we can evaluate that the technique is green, and even the use of toluene in a small volume of 3 μL did not detract from the evaluation. Figure 4 shows the results of the method evaluation with AGREE.

The assessment result according to AGREEprep is 71%. The limitations of the proposed method are as follows: the sample preparation is performed in the laboratory after sample collection and transportation, which affects the overall speed of sample preparation, and the use of the extraction solvent. Figure 4B shows the results of the method evaluation with AGREEprep.

According to the AESA evaluation, the developed method reached 90 points. According to the authors of AESA [35], the number of points above 75 is rated as an excellent green method. The biggest shortcomings in the evaluations were the work with toluene and the danger associated with it, as well as the large volume of the analyzed sample. Another focus of the extraction technique on even greater greenness can be the automation of the technique and the use of greener solvents than toluene. Table 4 shows the results of the method evaluation according to AESA.

### 2.6. Comparison of the Developed Method

The results obtained using the developed SDME-GC-μECD method were consistent with those from other sample preparation techniques, including MEPS, SPME, directly suspended solidified floating organic droplet microextraction (DS-SFOD), and SDME, and closely aligned with previously published data [13,16,22,39]. The developed method shows LOD and LOQ comparable or lower to those of existing approaches. Notably, it employs a minimal amount of sample and extraction solvent volume and multi-element analysis. These key advantages highlight the suitability for the determination of explosives. A comparative analysis between the proposed method and other relevant analytical techniques for explosive enrichment is presented in Table 5.

The table also lists actual findings by other authors. Actual findings fall within the concentration range presented in this paper.

## 3. Experimental

### 3.1. Chemicals and Reagents

All individual standards of nitro compounds were purchased from different sources (Dr. Ehrenstorfer GmbH, Augsburg, Germany) and provided by the Forensic Institute, Bratislava, Slovakia with purity higher than 95.66%. Stock standard solutions of the individual nitro compound, at a concentration of 1 mg/mL, were prepared by weighing the nitro compound and dissolving it in 10 mL of methanol (VWR Chemicals, Missouri City, MO, USA). The working standard solution of nitro compounds was prepared in methanol by dilution and stored at 4 °C. The working solution contained 9 nitro compounds at a concentration of 0.1 ng/μL. The calibration standards were prepared via additional dilution with toluene (Merck KGaA, Darmstadt, Germany). Solvents with different polarities, such as acetone, chlorobenzene, chloroform (all from Centralchem, Bratislava, Slovakia), dichloromethane, n-pentane, n-hexane and ethyl-acetate (all from Merck KGaA, Darmstadt, Germany), and acetonitrile (VWR Chemicals, Missouri City, MO, USA), were used as dilution solvents and for the preparation of working solutions. To obtain the optimum pH, citric acid (Mikrochem, Pezinok, Slovakia) and potassium hydroxide (Chemapol, Bratislava, Slovakia) were used. Sodium chloride (Centralchem, Bratislava, Slovakia) was added to optimize the addition of salt.

### 3.2. Samples and Procedures

The real tap water sample was collected in Bratislava, and seawater samples were taken from two sampling points on the island of Pag, Adriatic Sea (Croatia); the model forensic rinse water sample was prepared by swabbing the surface of the laboratory equipment with water moistened cotton swabs, thus imitating the real forensic sample collection at the site after an explosion. Real water samples were transferred to the analytical laboratory and stored in 1 L amber glass bottles in the fridge at 4 °C until DI-SDME-GC-ECD analysis.

The technique of DI-SDME was used to extract the analytes from the aqueous samples into the extract. A 10 μL microsyringe containing 3 μL of the extraction solvent was inserted into a 2 mL water sample contained in a 4 mL vial and subjected to magnetic stirring. The extraction solvent was ejected from the tip of the microsyringe, forming a drop on the microsyringe tip, and sample mixing was started. After the extraction time, the mixing machine was turned off, and the microdroplet was drawn back into the syringe and was injected into the GC system.

### 3.3. Instrumental Analysis and Data Evaluation

Chromatographic analyses were carried out on a gas chromatograph from Agilent Technologies, 6890N (Agilent Technologies, Little Falls, DE, USA). The chromatograph was equipped with a µECD (electron capture detector) and a split/splitless injector. Analytes were separated on a narrow-bore CP Sil 8 CB column with a stationary phase containing a 5% phenyl-polydimethylsiloxane polymer (15 m × i.d. 150 µm × 0.15 µm film thickness). SDMEs were performed on a magnetic stirrer LLG-uniSTIRRER 7. Extracts were injected in splitless mode using a 10 µL microsyringe. Hydrogen (4.0 Linde Technoplyn; Bratislava, Slovakia) was used as carrier gas at a constant flow rate of 1 mL/min. The injector temperature was set at 200 °C and used in splitless mode. The separations were carried out under temperature-programmed conditions. The initial temperature of the chromatographic oven was set at 90 °C and held for 1 min; subsequently, the temperature was increased to 130 °C at a 15 °C/min rate and held for 1 min. After that, the temperature was increased to 160 °C at a rate of 7 °C/min and held for 1 min, followed by a rate of 60 °C/min until 250 °C and held for 1 min. The analysis run time was 12.45 min. The detector temperature was set at 250 °C; nitrogen (5.0 Linde Technoplyn; Bratislava, Slovakia) was used as the makeup gas at a constant flow of 60 mL/min. The pH values of the solutions were measured on a Cyberscan pH 510 pH meter (Eutech Instruments, Vernon Hills, IL, USA). Sartorius Analytic MC1 scales (Sartorius, Göttingen, Germany) were used for weighing standards.

### 3.4. Method Validation

Deionized water, tap water, seawater, and model forensic rinse water samples were used as blanks for the preparation of matrix-matched standard solutions in the recovery study. The spiked standard solutions were prepared by adding working solutions of the respective nitro compounds using the optimized DI-SDME method.

#### 3.4.1. Repeatability, Linearity, Limit of Detection, and Limit of Quantification

RSD values were used to evaluate the repeatability of the method. Each extraction was repeated three times. The errors were calculated based on the standard deviation. The linearity of the method was evaluated using matrix-matched calibration curves through the spiking of blank sample extracts in the range from 0.5 to 250 μg/L. To evaluate the linearity, the coefficient of determination R^2^ was calculated. The limit of detection (LOD) and the limit of quantification (LOQ) were calculated from the slope of the calibration curve.

LOD = (3.3 ∗ standard deviation of the blank/slope of the calibration curve)(1)

LOQ = 3.3 ∗ LOD(2)

#### 3.4.2. Matrix Effects

The matrix effect was studied by calculating the matrix factor (MF) for each nitro compound. The MFs were calculated by comparing the peak area of the analyte in the matrix-matched solution vs. the peak area of the nitro compound in distilled water. The matrix-matched solution was prepared by using 3 μL of the matrix extract and 1 μL of the standard solution in toluene of known concentration.

MF = (peak area in matrix-matched solution/peak area in extract from distilled water − 1) ∗ 100(3)

## 4. Conclusions

This paper presents a broad study on the development of a green, environmentally friendly SDME method, with minimal consumption of solvents, energy, and waste generation. Eight nitro compounds were monitored in the water samples. Analyses were carried out using GC-μECD, and samples of deionized water and real water samples were analyzed. As part of method validation, linearity, precision, the limit of detection, and the limit of quantification were evaluated. The developed DI-SDME method can be reliably used for the analysis of selected analytes in water samples of monitored matrices. Matrix effects were evaluated, demonstrating that the matrix had an impact on the calibration process, and matrix-matched calibration is recommended. The greenness of the developed method was also evaluated with these techniques: AGREE (analytical greenness calculator software), AGREEprep, and the AESA method. With AGREE, the method reached 70%, while with AGREEprep, it reached 71%, and with AESA, the proposed method reached 90 points and was evaluated as an excellent green analysis.

## Figures and Tables

**Table 1:** Linearity, LOD, and LOQ for deionized, tap, sea, and forensic rinse water matrices.

Analyte	Deionized Water Matrix			Tap Water Matrix			Seawater Matrix			Forensic Rinse Water Matrix		
	Linearity R^2^	LOD (μg/L)	LOQ (μg/L)	Linearity R^2^	LOD (μg/L)	LOQ (μg/L)	Linearity R^2^	LOD (μg/L)	LOQ (μg/L)	Linearity R^2^	LOD (μg/L)	LOQ (μg/L)
NB	0.9973	0.02	0.08	0.9989	0.01	0.04	0.9991	0.01	0.02	0.9926	0.05	0.16
2-NT	0.9984	0.05	0.15	0.9993	0.06	0.19	0.9992	0.02	0.08	0.9965	0.03	0.11
3-NT	0.9961	0.08	0.28	0.9986	0.06	0.19	0.9992	0.01	0.03	0.9965	0.05	0.15
4-NT	0.9973	0.09	0.31	0.9974	0.02	0.07	0.9990	0.02	0.07	0.9611	0.11	0.38
1,3-DNB	0.9992	0.03	0.10	0.9986	0.01	0.05	0.9985	0.03	0.10	0.9873	0.05	0.17
1,2-DNB	0.9992	0.01	0.03	0.9946	0.01	0.03	0.9975	0.01	0.03	0.9820	0.06	0.21
2,4-DNT	0.9982	0.02	0.07	0.9975	0.02	0.06	0.9985	0.03	0.11	0.9848	0.06	0.19
TNT	0.9994	0.05	0.18	0.9977	0.01	0.05	0.9974	0.01	0.03	0.9620	0.09	0.31

Notes: R^2^—determination coefficient, LOD—limit of detection, and LOQ—limit of determination. Analyte name abbreviations: NB—Nitrobenzene, 2-NT—2-Nitrotoluene, 3-NT—3-Nitrotoluene, 4-NT—4-Nitrotoluene, 1,3-DNB—1,3-Dinitrobenzene, 1,2-DNB—1,2-Dinitrobenzene, 2,4-DNT—2,4-Dinitrotoluene, and TNT—Trinitrotoluene.

**Table 2:** Recovery and relative standard deviation for deionized, tap, sea, and forensic rinse water matrices.

Analyte	Deionized Water Matrix		Tap Water Matrix		Seawater Matrix		Forensic Rinse Water Matrix	
	Recovery (%)	RSD (%)	Recovery (%)	RSD (%)	Recovery (%)	RSD (%)	Recovery (%)	RSD (%)
NB	74.14	15.59	90.48	10.31	71.11	14.61	69.04	15.78
2-NT	73.47	26.03	81.78	11.86	63.94	23.59	35.65	51.06
3-NT	107.93	22.73	115.20	24.64	99.88	20.34	30.51	30.06
4-NT	73.90	23.16	91.89	28.53	62.15	24.81	36.98	4.20
1,3-DNB	64.51	24.80	101.11	17.61	61.99	13.52	52.60	2.17
1,2-DNB	62.16	23.90	101.63	20.78	63.49	17.87	57.79	14.68
2,4-DNT	64.63	25.09	106.26	19.54	63.56	9.86	63.39	21.24
TNT	56.46	24.87	100.12	20.25	60.01	14.31	101.61	48.56

Notes: RSD—relative standard deviation. Analyte name abbreviations: NB—Nitrobenzene, 2-NT—2-Nitrotoluene, 3-NT—3-Nitrotoluene, 4-NT—4-Nitrotoluene, 1,3-DNB—1,3-Dinitrobenzene, 1,2-DNB—1,2-Dinitrobenzene, 2,4-DNT—2,4-Dinitrotoluene, and TNT—Trinitrotoluene.

**Table 3:** Matrix factors of individual analytes in different types of water samples.

Analyte	MF in Tap Water (%)	MF in Seawater (%)	MF in Forensic Rinse Water (%)
NB	−31.13	18.64	−28.51
2-NT	−25.83	33.40	−23.84
3-NT	−31.69	18.61	−36.30
4-NT	−24.40	36.24	−55.52
1,3-DNB	−52.15	11.82	−2.16
1,2-DNB	−50.67	5.62	−0.89
2,4-DNT	−50.66	7.56	−0.93
TNT	−39.27	2.58	−0.91

Notes: MF—matrix factor. Analyte name abbreviations: NB—Nitrobenzene, 2-NT—2-Nitrotoluene, 3-NT—3-Nitrotoluene, 4-NT—4-Nitrotoluene, 1,3-DNB—1,3-Dinitrobenzene, 1,2-DNB—1,2-Dinitrobenzene, 2,4-DNT—2,4-Dinitrotoluene, and TNT—Trinitrotoluene.

**Table 4:** Evaluation of the greenness of the method according to AESA.

Procedure Parameters	Hazard	Penalty Points
Reagents	Toluene (3 μL)	6
Energy	GC-ECD	1
	Magnetic stirrer	0
Occupational hazard	Toluene vapors	3
Waste	Water sample (0 mL)	0
Sum	100 − 10 =	90

**Table 5:** Comparison of the developed method with methods reported earlier.

Sample Matrix	Analytes	Sample Preparation	Extraction Parameters	Instrumental Method	Recovery	LOD (LOQ)	Real Finds	References
Distilled and well water	2-CA, 2,5-DCA, 2-NT, 3-NT, 4-CNB, 2,5-DCNB, 3,4-DCNB	SPME	Sample size: 1.5 mL Time of extraction: 45 min Desorption time: 3 min Desorption temperature: 250 °C	GC-FID	73–119%	1–10 μg/L 30–50 μg/L	2-CA 0.06 mg/L 0.63 mg/L 2,5-DCA 0.36 mg/L 3,4-DCNB 0.08 mg/L 9.42 mg/L 2-NT, 3-NT, 4-CNB, 2,5-DCNB Not detected	[13]
Underground water	NB, TNT, Tetryl, 1,3,5-TNB, 4-ADNT, 1,3-DNB, 2,4-DNT, 2,6-DNT, 2-NT, 3-NT, 4-NT	MEPS	Sample size: 10 × 50 μL Washing solvent: 50 μL of water Elution solvent: 30 μL of MeOH	GC-MS	77.5–99.2%	0.014–0.828 pg/mL 0.046–2.732 ng/mL	NB 1.03 ng/mL 2-NT 0.38 ng/mL 3-NT 0.81 ng/mL 2,4-DNT 0.22 ng/mL	[16]
Surface, tap, and well water	TNT, 2,4-DNT, 2,6-DNT	DS-SFOD	Sample size: 10 mL Extraction solvent: 40 μL of DES (Decanoic acid–borneol) Time of extraction: 30 min	HPLC-UV	89–102%	0.14–0.19 μg/L 0.5–0.6 μg/L	˂LOD	[22]
Well and underground water	HMX, RDX, PETN, TNB, CL-20, Tetryl, TNT, 2,6-DNT, 3-NT, 2,4-DNT, 2-NT	d-SDME	Sample size: 10 mL Extraction temperature: 30 °C Extraction solvent: 50 μL (ferrofluid) Time of extraction: 30 min Dissolving solution: 500 μL of Acetonitrile	HPLC-UV	88–103.7%	0.22–0.91 μg/L 0.73–3 μg/L	RDX 2.3 μg/L TNB 0.7 μg/L 6.4 μg/L TNT 2.5 μg/L 12.4 μg/L 2,6-DNT 0.9 μg/L 9.8 μg/L	[39]
Deionized, tap, sea, and forensic rinse water	NB, 2-NT, 3-NT, 4-NT, 1.3-DNB, 1.2-DNB, 2.4-DNT, TNT	SDME	Sample size: 2 mL Extraction temperature: 22 °C Extraction solvent: 3 μL Toluene Time of extraction: 35 min	GC-μECD	57–115%	0.01–0.11 μg/L 0.03–0.38 μg/L	˂LOD	This method

Notes: LOD—limit of detection, LOQ—limit of determination, DS-SFOD—directly suspended solidified floating organic droplet microextraction, SPME—solid phase microextraction, MEPS—microextraction by packed sorbent, d-SDME—directly suspended droplet microextraction, and SDME—single-drop microextraction. Analyte name abbreviations: 2-CA—2-Chloroaniline, 2,5-DCA—2,5-Dichloroaniline, 2-NT—2-Nitrotoluene, 3-NT—3-Nitrotoluene, 4-CNB—4-Chloronitrobenzene, 2,5-DCNB—2,5-Dichloronitrobenzene, 3,4-DCNB—3,4-Dichloronitrobenzene, NB—Nitrobenzene, TNT—2,4,6-Trinitrotoluene, Tetryl—2,4,6-Trinitrophenyl-Nmethylnitramine, 1,3,5-TNB—1,3,5-Trinitrobenzene, 4-ADNT—4-Amino-2,6-dinitrotoluene, 1,3-DNB—1,3-Dinitrobenzene, 2,4-DNT—2,4-Dinitrotoluene, 2,6-DNT—2.6-Dinitrotoluene, 4-NT—4-Nitrotoluene, HMX—Cyclotetramethylene tetranitramine, RDX—1,3,5-Trinitroperhydro-1,3,5-triazine, PETN—Pentaerythritol tetranitrate, TNB—1,3,5-Trinitrobenzene, CL-20—Hexanitrohexaazaisowurtzitane, 1,3-DNB—1,3-Dinitrobenzene, and 1,2-DNB—1,2-Dinitrobenzene.

## Data Availability

All relevant data are included in the paper.
